# Enhanced Optoelectronic Conversion Efficiency of CdSe/ZnS Quantum Dot/Graphene/Silver Nanowire Hybrid Thin Films

**DOI:** 10.1186/s11671-016-1606-3

**Published:** 2016-09-06

**Authors:** Bo-Tau Liu, Kuan-Han Wu, Rong-Ho Lee

**Affiliations:** 1 Department of Chemical and Materials Engineering, National Yunlin University of Science and Technology, 123 Univ. Rd., Sec. 3, Douliou, Yunlin, 64002 Taiwan; 2 Department of Chemical Engineering, National Chung Hsing University, Taichung, 40227 Taiwan, Republic of China

**Keywords:** CdSe/ZnS, Quantum dot, Graphene, Silver nanowire, Quenching, Optoelectron

## Abstract

In this study, we prepared the reduced graphene oxide (rGO)-CdSe/ZnS quantum dots (QDs) hybrid films on a three-layer scaffold that the QD layer was sandwiched between the two rGO layers. The photocurrent was induced by virtue of the facts that the rGO quenched the photoluminescence of QDs and transferred the excited energy. The quenching mechanism was attributed to the surface energy transfer, supported in our experimental results. We found that the optoelectronic conversion efficiency of the hybrid films can be significantly improved by incorporating the silver nanowires (AgNWs) into the QD layer. Upon increasing AgNW content, the photocurrent density increased from 22.1 to 80.3 μA cm^−2^, reaching a near 3.6-fold enhancement compared to the pristine rGO-QD hybrid films. According to the analyses of photoluminescence spectra, shape effect, and electrochemical impedance spectra, the enhancement on the optoelectronic conversion efficiency arise mainly from the strong quenching ability of silver and the rapid electron transfer of AgNWs.

## Background

Due to the confinement of the charge carriers in three spatial dimensions, quantum dots (QDs) display extraordinarily optoelectronic properties and tunable band gap. Over the past decade, QDs have been widely studied on the application of solar cells [1, 2], sensors [3], light emitters [4, 5], and bioassays [6]. Recently, many studies revealed that the excited energy of QDs could be transfer effectively to graphene because of the high conductivity and luminescence quenching ability of graphene [7–11]. In general, the quenching possible mechanism can be ascribed to the following routes: Forster resonance energy transfer, surface energy transfer, and photo-induced electron transfer [9]. Some studies have shown experimentally that the quenching of QDs by graphene was assigned to surface energy transfer [10, 12]. The rate of surface energy transfer and the rate of Forster resonance energy transfer are inversely proportional to the fourth and sixth power of the distance between donors and acceptors, respectively. Therefore, the surface energy transfer occurs in a larger range than the Forster resonance energy transfer. At a relatively long distance, the surface energy transfer is more efficient than the Forster resonance energy transfer. The highly effective charge transfer can avoid the recombination of excited electrons and holes and is in favor of the optoelectronic conversion. Therefore, the QD-graphene system has been applied to improve pollution detection [13, 14], light-harvesting devices [15–19], QD-sensitized solar cells [20–22], bioassays [23], etc.

In fact, the photoluminescence (PL) suppression of QDs also occurs in the presence of metals due to the Forster resonance energy transfer [24]. Unlike graphene, the metal nanostructural surfaces, nanoparticles, or nano-holes not only quench but also enhance the PL of QDs through the excitation of localized surface plasmon resonance (LSPR) of metal nanostructures, which amplifies the local electric field to alter the optical properties of QDs [25–31]. As a result, both the PL quenching and enhancement are observed after the excitons of QDs coupling with LSPR of metal nanostructures. Because the Forster energy transfer is a shorter range effect than the enhanced electromagnetic field, the PL quenching will be weakened with distance and much faster than the LSPR enhancement. At longer distance, the PL enhancement decrease gradually [32–35]. The distance of the QDs from metal nanostructural surfaces affects the competition between enhancement and quenching. Moreover, the LSPR absorption characteristic depends strongly on the size, shape, and coupling of metal nanoparticles and the dielectric properties of their surrounding medium [36–38]. As a result, the effect of nanometals on optoelectronic conversion of QDs is complicated and undetermined.

In this study, we used reduced graphene oxides (rGOs) and CdSe/ZnS QDs to fabricate rGO-QD-rGO sandwich-structure films. The sandwich structure is willing to alleviate the deterioration of QDs in surroundings by virtue of the covering of graphene. We found that the optoelectronic conversion efficiency of the QD-graphene system was significantly improved by incorporating silver nanowires (AgNWs) into the QD layer. The optimal composition for the hybrid films was analyzed and discussed. The incorporation of silver nanoparticles (AgNPs) and silver nanorods (AgNRs) was also done in order to realize the mechanism of enhancement of AgNWs.

## Methods

### Preparation of Water-Soluble CdSe/ZnS Core-Shell QDs

Water-soluble CdSe/ZnS core-shell QDs were synthesized as reported previously [39]. Briefly, solvent-based CdSe/ZnS QDs dispersed in chloroform were synthesized by the solvothermal methods: CdSe core and ZnS shell were prepared at 290 °C for 5 min and at 220 °C for 1 min, respectively. Excess 3-mercaptopropionic acid (MPA; Sigma-Aldrich) was added into 10 wt.% KOH methanol solution, and the mixture was violently stirred. The as-prepared CdSe/ZnS chloroform solution was added into the MPA solution in the volume ratio of 2:1. After 5-min mixing, the QDs in the suspension were precipitated with the addition of acetone. The QDs were purified through centrifugation (9000 rpm, 10 min), decanting the supernatant, and redispersing the precipitate with methanol. Finally, the precipitate was redispersed in water, resulting in MPA-capped CdSe/ZnS QD aqueous solution.

### Preparation of AgNPs, AgNRs, and AgNWs

A 0.17 g of AgNO_3_ (Showa) and 0.17 g of polyvinylpyrrolidone (PVP, Acros) were mixed in 10 mL of water. A 0.028 g of NaBH_4_ (Alfa Aesar) was then added rapidly into the AgNO_3_ aqueous solution. After 10 min, the resulting solution was precipitated by acetone and then redispersed with water several times, resulting in the AgNPs.

AgNRs (to be more exact, the nanorods are Au-Ag core-shell structure.) were synthesized using the seed-mediated growth method as reported by Zhou et al. [40]. Briefly, 0.4 mL of AgNO_3_ (0.01 M), 10 mL of HAuCl_4_ (Fluka, 0.01 M) and 10 mL of cetyltrimethylammonium bromide (C_16_TAB, Sigma-Aldrich, 0.1 M) were mixed. Then, 0.32 mL of ascorbic acid (AA; Sigma-Aldrich, 0.1 M), 0.8 mL of HCl (1 M), and 96 μL of the seed solution were added into the mixture sequentially. The mixture was stirred rigorously for 1 min and then undisturbed for 6 h. A 2 mL of the mixture was washed three times with cetyltrimethylammonium chloride (CTAC; Sigma-Aldrich, 0.1 M) through centrifugation and then re-dispersed in 10 mL of CTAC (80 mM). The resultant solution was reacted with 0.5 mL of AA (100 mM) and 0.17 mL of AgNO_3_ (0.01 M) at 60 °C for 3 h, resulting in the AgNRs.

AgNWs were synthesized as reported previously [41]. Briefly, 20 μL of AgNO_3_ (1 M) was added into the mixture of 36 mL of PVP (0.3 M) and 80 μL of NaCl (0.2 M) at 160 °C. A 4 mL of AgNO_3_ (1 M) was then added slowly into the mixture using a peristaltic pump. The solvent of all the above-mentioned solutions is ethylene glycol (EG). When the color of the mixture turned into a misty auburn, all of the residual AgNO_3_ solution was poured into the mixture at once. After the color of the solution turned into silver-whitish, the products were washed three times with ethanol through centrifugation, resulting in the AgNWs.

### Preparation of the rGO-QD, rGO-QD/AgNW, rGO-QD/AgNR, and rGO-QD/AgNP Sandwich Structures

Indium tin oxide (ITO) glass was rinsed with acetone and de-ionized water through ultrasonication. The cleaned ITO glass was immersed into 10 wt.% 3-aminopropyltrimethoxysilane (APTS) aqueous solution and then dried at 70 °C. Various amounts (100, 300, 500, and 700 μL) of GO solution (4 mg/mL), fabricated by the modified Hummers method as reported in the previous work [42], were diluted to 2 mL. A 100 μL of CdSe/ZnS QD solution was diluted to 800 μL. GO, QD, and GO was sequentially spin-coated on the APTS-treated ITO glass substrates (2 × 2 cm). The hybrid film was annealed under N_2_ atmosphere at 200 °C for 15 min and then immersed in 10 wt.% hydrazine solution at 80 °C for 30 min, resulting in the rGOx-QD hybrid film, where *x* denotes x00-μL GO solution was added. The rGOx-QD/AgNWy hybrid films were prepared as that of the rGOx-QD ones, except that various amount of AgNW solution (100, 300, 500, and 700 μL) were mixed with the QD solution, where *y* indicates y00-μL AgNW solution was added. To realize the effect of silver shape on the enhancement of optoelectronic conversion efficiency of rGO-QD hybrid films, the AgNWs were replaced by AgNRs and AgNPs individually on the same amount to prepare the hybrid films, denoting as rGOx-QD/AgNR and rGOx-QD/AgNP, respectively.

### Measurements

Particle size and morphology of the as-prepared CdSe/ZnS QDs, AgNPs, and AgNRs were examined using a field-emission scanning-electron microscope (SEM; JSM-7401F, JEOL) and a high-resolution transmission electron microscope (TEM; JEM-2010, JEOL). The absorption spectra of CdSe/ZnS QDs and hybrid films were measured using a UV-Vis spectrophotometer (Lambda 850, PerkinElmer). PL spectra of CdSe/ZnS QDs and their hybrid films were measured using fluorescence spectrophotometer (LS-55/45, PerkinElmer). The size and morphology of the GO and AgNWs were characterized using optical microscopy (OM; M835, M&T Optics). Optoelectronic conversion of the hybrid films was measured through a photoelectrochemical bath: the electrolyte solution was Na_2_SO_3_ (0.35 M) and Na_2_S (0.24 M) in water, and the hybrid film (2 × 2 cm), a Pt wire, and a Ag/AgCl electrode were used as the working, counter, and reference electrodes, respectively. The photocurrent of the working electrode and the electrochemical impedance spectra (EIS) over the frequency range of 50 mHz–100 kHz with a potential perturbation of 10 mV were measured using an electrochemical workstation (Zennium, Zahner) under irradiation of a 75-W halogen lamp with 2-cm interval between the lamp and the working electrode.

## Results and Discussion

**Fig. 1:**
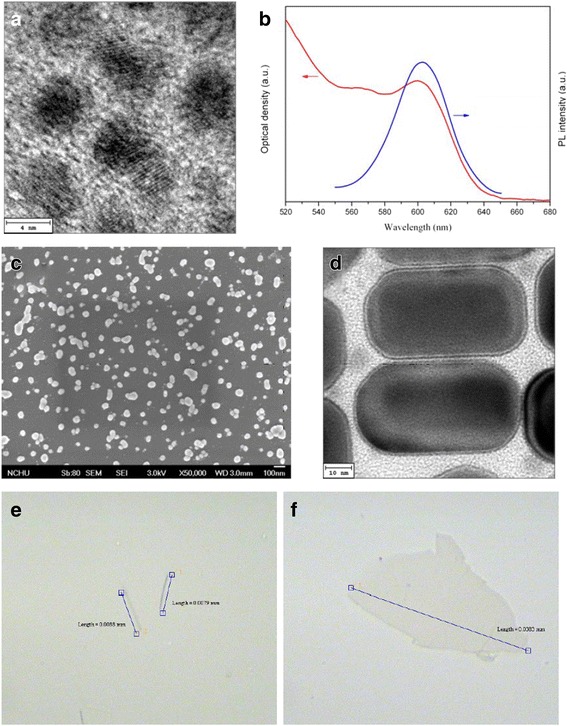
**a** TEM image and **b** PL emission and UV-Vis absorption spectra of the as-prepared CdSe/ZnS QDs. **c** SEM image of the as-prepared AgNPs. **d** TEM image of the as-prepared AgNRs. **e** OM image of the as-prepared AgNWs. **f** OM image of the as-prepared GO

**Fig. 2:**
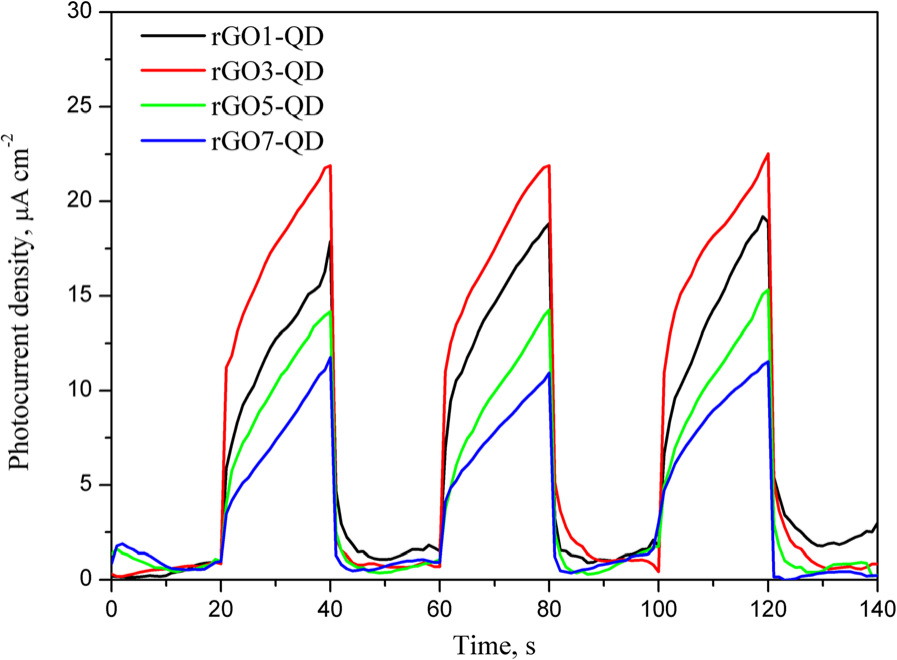
Photocurrent density-time curves of the rGOx-QD sandwich-structure hybrid films with various rGO amounts under on/off-cycle light irradiation

Figure 1 displays the images of the prepared QDs, AgNPs, AgNRs, AgNWs, and GO. The particle size of the as-prepared CdSe/ZnS QDs is over 4~5 nm (Fig. 1a), with a PL emission wavelength at 603 nm and an absorption peak at 600 nm (Fig. 1b). The diameter of AgNPs, the length of AgNRs, and the length of AgNWs are about 52 nm, 68 nm, and 8.5 μm referring to Fig. 1c–e, respectively. The size of the as-prepared GO is about tens micrometer. The rGO-CdSe/ZnS QD sandwich structure revealed that the photon could be inverted into the current by virtue of the fact that rGO quenches the PL of QDs. Figure 2 shows that the photocurrent increases with small increments of rGO (100 to 300 μL). Since the excess rGO stacks on the top of the lower rGO layer rather than directly contacts QDs, the photocurrent raise supports the argument that graphene can quench the PL of QDs at a relatively long distance, namely, the surface energy transfer. With the further rGO addition, the photocurrent decreased because incident light was absorbed by numerous rGO and thereby its intensity and dose reduce to excite the QDs.

**Fig. 3:**
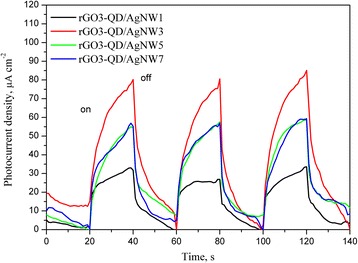
Photocurrent density-time curves of the rGO3-QD/AgNWy sandwich-structure hybrid films with various AgNW amounts under on/off-cycle light irradiation

**Fig. 4:**
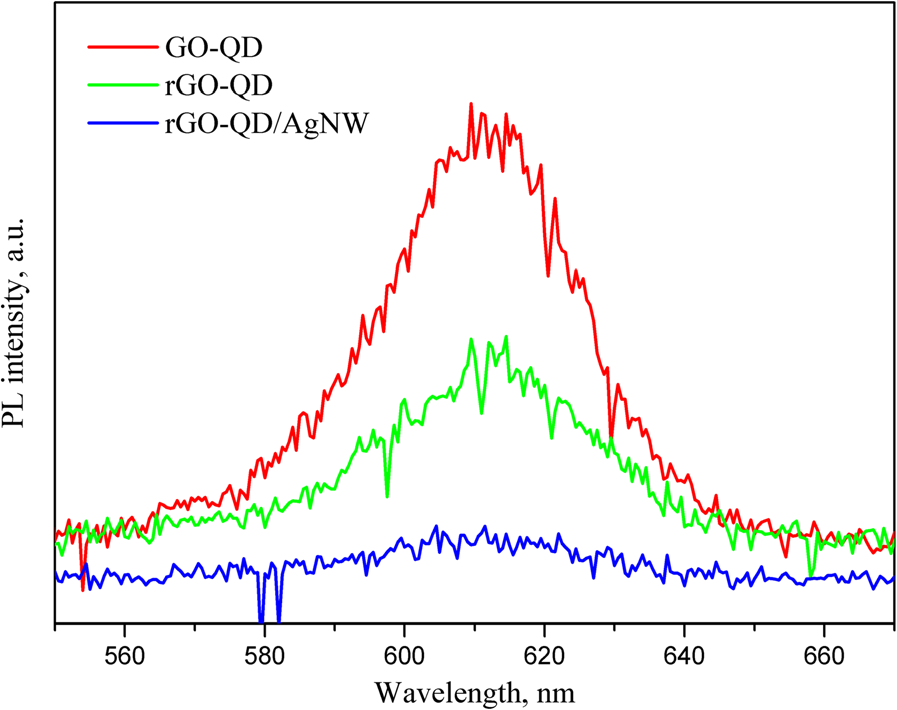
PL spectra of the GO-QD, rGO-QD, and rGO-QD/AgNW hybrid films under 350-nm excitation

**Fig. 5:**
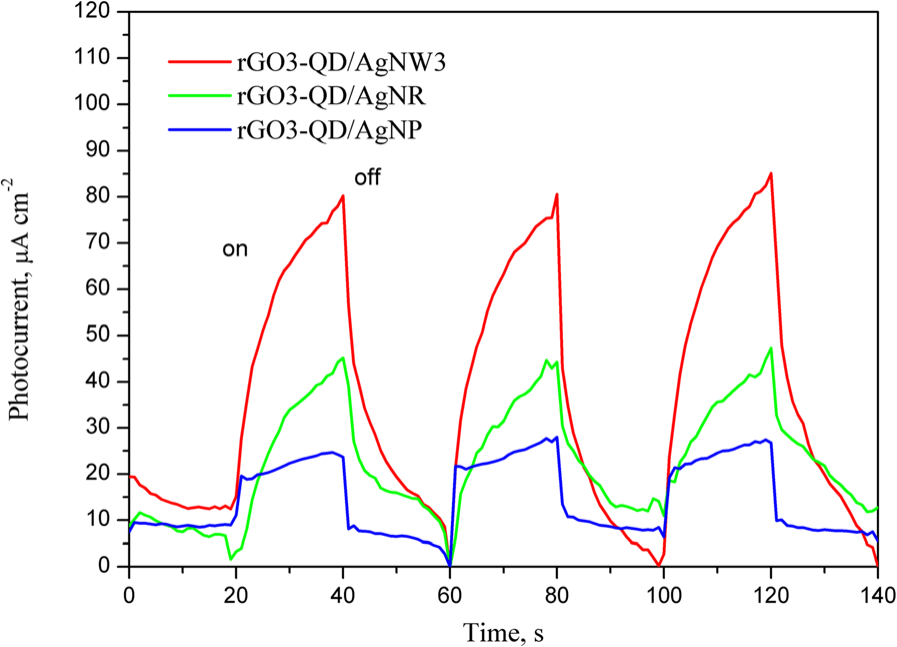
Photocurrent density-time curves of the rGO3-QD/AgNW3, rGO3-QD/AgNR, and rGO3-QD/AgNP hybrid films under on/off-cycle light irradiation

Besides exciting and quenching the PL of QDs, AgNWs may absorb and scatter the incident light by the localized surface plasmon resonance and the large diameter [41], respectively. Therefore, the effect of AgNWs on optoelectronic conversion efficiency of QDs is still vague. We incorporated AgNWs into the QD layer and found the AgNW incorporation can enhance significantly the photocurrent, shown in Fig. 3. While the addition of AgNWs changed from 0 to 300 μL, the photocurrent density increased from 22.1 to 80.3 μA cm^−2^, a near 3.6-fold enhancement. However, too much AgNW incorporation reduced the photocurrent enhancement as a result of the high extinction coefficient and the large scattering effect of AgNWs. In order to realize the mechanism of AgNW enhancement on the photocurrent, the PL spectra of rGO-QDs with/without AgNWs were measured (Fig. 4). Although rGO shows the ability of quenching the PL, the AgNW incorporation can enhance the suppression on the PL, being more efficient to transfer the exciton energy. We evaluated the influence of the various shapes of silver (AgNPs, AgNRs, AgNWs) on the optoelectronic conversion efficiency. Figure 5 shows the photocurrent response of the rGO3-QD/AgNW3, rGO3-QD/AgNR, and rGO3-QD/AgNP hybrid films. The photocurrent density increases in the following sequence: rGO3-QD/AgNP < rGO3-QD/AgNR < rGO3-QD/AgNW3. Referring to Fig. 1, the diameter of AgNPs, the length of AgNRs, and the length of AgNWs follow the order: AgNP (52 nm) < AgNR (68 nm) < AgNW3 (8.5 μm). The order of their magnitude is the same as that of their photocurrent density; the AgNWs exhibit the maximum length and the highest photocurrent density. Accordingly, we infer that the AgNW enhancement on the optoelectronic conversion efficiency may arise from not only the strong quenching nature of silver but also the rapid electron transfer along the axial direction.

**Fig. 6:**
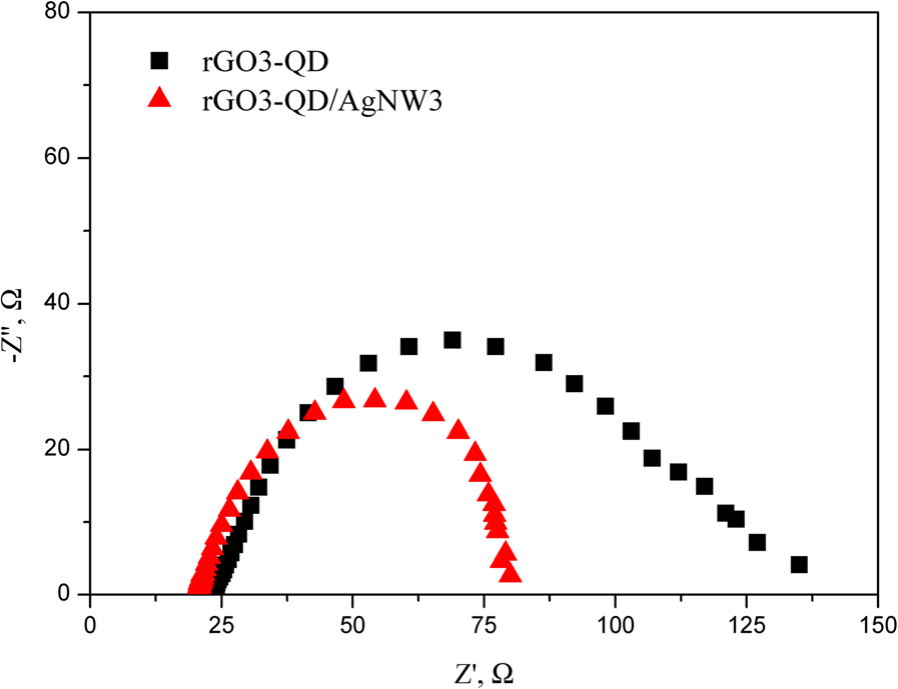
Nyquist plots of the electrochemical impedance spectra of rGO3-QD and rGO3-QD/AgNW3

Figure 6 displays Nyquist plots of the EIS for the rGO3-QD and rGO3-QD/AgNW3. Both the plots exhibit a semicircle, indicating the charge transfer resistance at the hybrid films. The rGO3-QD/AgNW3 (58.4 Ω) shows lower impedance than the rGO3-QD (88.4 Ω). The electron lifetime, which is inverse to the reaction rate constant for charge recombination, can be determined from the meddle-frequency peak in the Nyquist plots. The rGO3-QD/AgNW3 (0.110 s) reveals the longer electron lifetime than the rGO3-QD (0.047 s). Accordingly, AgNW incorporation decreases the charge transfer resistance and the probability of charge recombination, resulting in the remarkable increase of photocurrent. The result is in good agreement with that in the analysis of shape effect (Fig. 5).

## Conclusions

The rGO-CdSe/ZnS QD hybrid thin films have been fabricated on a sandwich scaffold. The optoelectronic conversion efficiency of the hybrid film was significantly enhanced by incorporating AgNWs into the QD layer. However, too low or high rGO or AgNW addition decreased the enhanced performance. Compared to AgNPs and AgNRs, the AgNWs displayed superior improvement on optoelectronic conversion. The optimal AgNW incorporation can result in a near 3.6-fold enhancement on the photocurrent density in comparison with the pristine rGO-QD hybrid film. We infer that the enhancement on the optoelectronic conversion efficiency may arise from the strong quenching ability of silver and the rapid electron transfer of AgNWs.
